# Mendelian randomization: estimation of inpatient hospital costs attributable to obesity

**DOI:** 10.1186/s13561-021-00314-2

**Published:** 2021-05-14

**Authors:** Katherine Dick, John E. Schneider, Andrew Briggs, Pascal Lecomte, Stephane A. Regnier, Michael Lean

**Affiliations:** 1 Avalon Health Economics, 26 Washington St. 2nd Floor, Morristown, NJ 07960 USA; 2 London School of Hygiene and Tropical Medicine, Keppel St, Bloomsbury, London, WC1E 7HT UK; 3 Novartis AG, WSJ - 210.15.30.23, CH-4056 Basel, Switzerland; 4 University of Glasgow, University Avenue, Glasgow, G12 8QQ Scotland

**Keywords:** Mendelian randomization, Obesity, Instrumental variables, Genetics, Economics, Healthcare utilization

## Abstract

**Background:**

Mendelian Randomization is a type of instrumental variable (IV) analysis that uses inherited genetic variants as instruments to estimate causal effects attributable to genetic factors. This study aims to estimate the impact of obesity on annual inpatient healthcare costs in the UK using linked data from the UK Biobank and Hospital Episode Statistics (HES).

**Methods:**

UK Biobank data for 482,127 subjects was linked with HES inpatient admission records, and costs were assigned to episodes of care. A two-stage least squares (TSLS) IV model and a TSLS two-part cost model were compared to a naïve regression of inpatient healthcare costs on body mass index (BMI).

**Results:**

The naïve analysis of annual cost on continuous BMI predicted an annual cost of £21.61 [95% CI £20.33 – £22.89] greater cost per unit increase in BMI. The TSLS IV model predicted an annual cost of £14.36 [95% CI £0.31 – £28.42] greater cost per unit increase in BMI. Modelled with a binary obesity variable, the naïve analysis predicted that obese subjects incurred £205.53 [95% CI £191.45 – £219.60] greater costs than non-obese subjects. The TSLS model predicted a cost £201.58 [95% CI £4.32 – £398.84] greater for obese subjects compared to non-obese subjects.

**Conclusions:**

The IV models provide evidence for a causal relationship between obesity and higher inpatient healthcare costs. Compared to the naïve models, the binary IV model found a slightly smaller marginal effect of obesity, and the continuous IV model found a slightly smaller marginal effect of a single unit increase in BMI.

## Background

The global prevalence of obesity has increased significantly since 1980 and represents a significant economic burden worldwide [1–5]. Analysis of Global Burden of Disease data (2015) estimated that 603.7 million adults were obese, representing 12% of adults globally [6]. According to the World Health Organization criteria used internationally and by the UK National Health Service (NHS), an adult with a body mass index (BMI) of 18.5 to 25.0 kg/m^2^ is considered to be of a normal weight. Individuals with a BMI of 25 to 30 kg/m^2^ are considered to be overweight, and individuals with a BMI at or above 30 kg/m^2^ are considered to be obese [7]. Obesity has a significant impact on health through secondary consequences such as type 2 diabetes, coronary heart disease, cancer, stroke, and depression [8–10].

Observational studies have established a positive correlation between elevated BMI and healthcare costs, but they cannot definitively establish causation because of the high likelihood of unobserved confounding factors and the possibility of reverse causation [1, 4, 11]. Randomized controlled trials may establish causation, but there are challenges to conducting randomized controlled trials in obesity [12–14]. Mendelian Randomization (MR) is a type of instrumental variable analysis that uses genetic variants as instruments to mitigate the effects of unobserved confounding factors in statistical models. In the case of this study, MR is used to evaluate the relationship between the risk factor of elevated BMI on the outcome of inpatient healthcare costs.

To our knowledge, at the time this study was designed, no published studies had investigated the link between obesity and healthcare costs using MR methods, though general instrumental variable analysis has been used extensively to investigate the relationship with obesity and other factors such as cancer and cardiovascular diseases [15–18]. A few studies have used general instrumental variable analysis to investigate the relationship between obesity and healthcare costs, but these studies did not use genetic variants as instruments. Cawley and Meyerhoefer [19] conducted an instrumental variable analysis of US Medical Expenditure Panel Survey data to determine that obesity increases annual medical costs by $2741. The study used the weight of a biological relative as an instrument for the weight of the primary subject. An Australian instrumental variables study on the impact of childhood obesity on healthcare costs found that obese individuals incurred healthcare costs $102.90 AUD greater than their normal weight counterparts. This study by Black et al. used a biological parent’s BMI as an instrument for the child’s BMI [20].

Our study aims to use MR to estimate the impact of obesity on annual inpatient healthcare costs in the UK using linked data from the UK Biobank and Hospital Episode Statistics (HES). The use of MR methods to investigate the relationship between obesity and healthcare costs is a novel approach, and this analysis was intended to fill a gap in the literature. A study using similar methods and conducted independently of ours was recently published, and reported marginal effects per additional unit of BMI ranging from £18.85 [95% CI: 9.05–28.65] to £21.22 [95% CI: 14.35–28.07] [21]. There are some key differences in the approach, including the use of a larger sample size, data over a longer follow-up period, and different modelling strategies, but the results of both studies suggest a significant causal relationship between obesity and inpatient healthcare costs. This study offers an applied example of the use of Mendelian Randomization in health economics research and is a useful independent replication of the recent results reported by Dixon et al.

## Methods

### Instrumental variable analysis

An instrumental variable, in this case a genetic variant, must fulfill three assumptions: 1) the instrument must be associated with the risk factor (relevance assumption); 2) the instrument must be associated with the outcome only through its association with the risk factor (exclusion-restriction assumption); and 3) the instrument must be independent of factors that may affect the outcome (independence assumption) [14, 22].

**Fig. 1:**
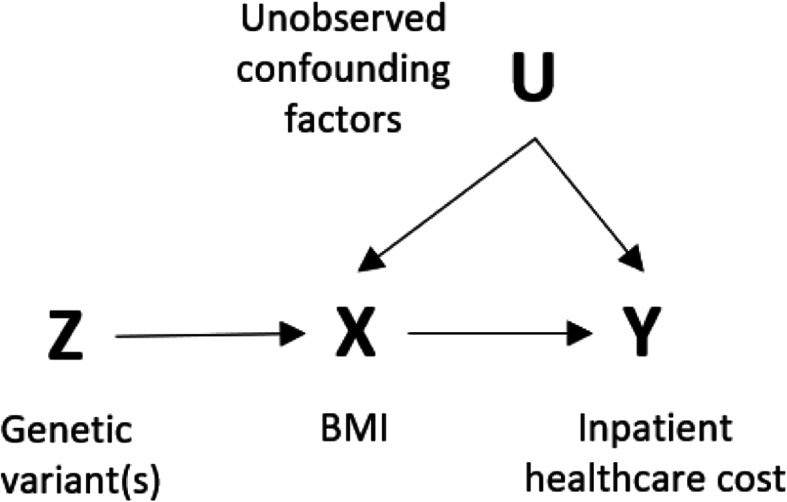
Directed acyclic graph (DAG) of relationship between instrumental variable Z, exposure X and outcome Y

Genes are randomly allocated, conditional on parental genes, according to Mendel’s laws of inheritance, and are therefore generally independent of external factors [14, 22]. The presence of obesity will not alter the genotype, so reverse causation is also not a concern [14, 23]. These characteristics make genetic variants viable instrumental variables. Figure 1 presents a directed acyclic graph that depicts the relationship between the instrument, exposure, and outcome.

### Data sources

The UK Biobank is a medical research database supported by the NHS. Between 2006 and 2010, the UK Biobank collected detailed demographics, health data and biological samples from over 500,000 participants between the ages of 40 and 69. The UK Biobank reports patient-level genotype data [24]. The UK Biobank is also linkable to inpatient admissions data from the HES database, which contains information on the diagnoses and procedures associated with each hospital admission [25]. These admissions are constructed in terms of hospital spells and episodes. A hospital spell represents the time from hospital admission to discharge. Within each hospital spell, subjects may experience several episodes of care, which are continuous periods of care under a single consultant. The linkage between the UK Biobank and HES records connects the genotype data necessary for MR analysis with the outcome of interest, healthcare resource use.

### Subjects

**Fig. 2:**
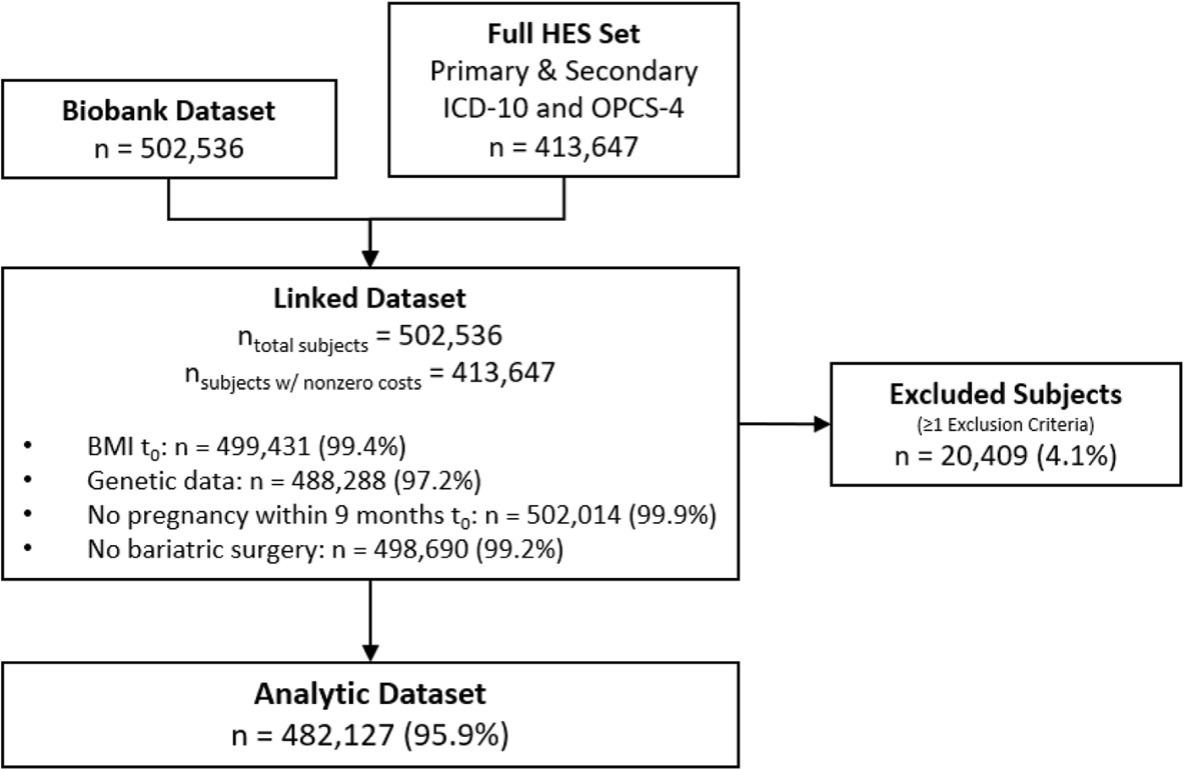
Flowchart of dataset construction and subject exclusion

This study included all UK Biobank subjects with data for age, gender, and baseline BMI measurements and selected genetic variants. The UK Biobank data was linked with HES records using a unique patient identifier. A small number of patients who received bariatric surgery were excluded from the analysis because of the potential for rapid weight loss with elevated inpatient healthcare costs, which may obscure the relationship between BMI and inpatient healthcare cost. Female subjects with a confirmed pregnancy within 9 months of the baseline BMI measurement were also excluded due to possible confounding from pregnancy-related weight gain. The final sample included 482,127 subjects. A flowchart depicting dataset construction and subject exclusions is presented in Fig. 2**.**

### Instrument

Genome-wide association studies (GWASs) are an invaluable resource for investigating diseases mediated by multiple genes. The objective of GWASs is to identify specific genetic variants, known as small-nucleotide polymorphisms (SNPs), that are associated with a particular trait or disease. Dozens of obesity-associated genetic variants have been identified in GWAS studies with low but significant explanatory power [26–28]. Alone, these variants would be weak instruments, but when combined into a genetic risk score (GRS), the variants can explain a greater amount of variance in BMI and reduce weak-instrument bias [29, 30].

A 2013 study by Belsky et al. synthesized data from 16 GWAS studies with the objective of developing a GRS to efficiently and effectively predict subjects’ predisposition toward obesity. SNPs were systematically chosen for inclusion in Belsky’s GRS based on both strength of association with obesity and the number of times the specific SNP was identified in different GWAS studies (i.e., replicability). The instrument was found to be a statistically significant predictor of BMI and obesity [31]. Belsky’s GRS formed the basis of the instrument used in this study, though not all of the specific SNPs were available in the UK Biobank data. In cases where the exact SNPs were unavailable in the genotyping platform implemented by the UK Biobank, SNPs on the same gene were chosen from the largest, most recent GWAS study included in Belsky’s analysis (i.e., Speliotes et al.) [27, 31]. Following the method employed by Belsky et al., each SNP was weighted according to its effect size and summed to generate the GRS.

MR analysis is only useful if the instrument is proven to be both valid and strong. Such an instrument does not violate any of three instrumental variable assumptions. The relevance assumption requires that the instrument (i.e. the GRS) be strongly associated with the exposure (i.e. BMI). The F-statistic is a common metric of instrument strength in MR analysis; typically, an F-statistic above 10 generally suggests a strong instrument [32]. The independence assumption requires that the instrument is not associated with any factors that may influence the outcome (i.e. inpatient healthcare cost) such as age, sex, ethnicity, or socioeconomic status. These factors generally have no direct effect on gene assignment, though systematic differences in allele frequency can occur in certain subpopulations, which is referred to as population stratification. This can be tested by linear regression of the instrument on each covariate [22, 29, 33].

The exclusion-restriction assumption requires that the GRS be associated with healthcare cost only through its association with BMI. Linkage disequilibrium and pleiotropy are both violations of this assumption. Linkage disequilibrium is a correlation between two genetic variants that occurs when traits are inherited together (i.e. not randomly), often in close proximity on a chromosome. Linkage disequilibrium violates the exclusion-restriction assumption if a variant in the GRS is in linkage disequilibrium with another variant that is associated with traits that also influence cost [14, 22]. For example, the assumption is violated if a variant in the GRS is in linkage disequilibrium with a variant associated with breast cancer, which is also associated with greater inpatient healthcare cost. Pleiotropy occurs when a genetic variant has multiple functions that may be related to the outcome variable [14, 22]. For example, the assumption is violated if a variant in the GRS is associated with both elevated BMI and increased risk of cardiovascular disease because cardiovascular disease also impacts inpatient healthcare cost. There is no direct test for these violations, but searching GWAS studies provides information on the variants’ associations with other genes and traits that could indicate violation by pleiotropy or linkage disequilibrium. The National Human Genome Research Institute’s (NHGRI) GWAS Catalog is a database of all known GWAS studies, which enables a search for all currently known gene associations [22, 34].

### BMI

According to the World Health Organization criteria used internationally and by the UK NHS, an adult with BMI of 18.5 to 25.0 kg/m^2^ is considered to be of a normal weight. Individuals with a BMI of 25 to 30 kg/m^2^ are considered to be overweight, and individuals with a BMI at or above 30 kg/m^2^ are considered to be obese [7]. Although BMI is a commonly used measure for obesity, it is an imperfect measure that does not distinguish between lean and fat mass [35, 36].

BMI may be treated as a continuous variable or as a categorical variable. The categorical variable tested in this analysis was binary; subjects below the threshold of 30 kg/m^2^ were categorized as non-obese, and subjects above the threshold were categorized as obese. All models in this analysis were executed twice, representing BMI as a continuous variable or as a binary variable.

### Inpatient healthcare costs

The linked HES data provides episode-level information about diagnoses, procedures, and hospital length of stay. This information was used to classify each episode of care by Healthcare Resource Group (HRG). HRGs are groupings of clinically similar diagnoses, procedures, and treatments that use similar resources and may be linked to a national unit cost [37]. An NHS application referred to as the HRG Reference Costs Grouper was used to process hospital data and appropriately assign HRGs to episodes of care [38]. Each HRG code was assigned a cost based on the 2017/2018 NHS National Schedule of Reference Costs [39]. In some cases, records were assigned two HRG codes, one of which was an “unbundled” code, which separately captures high-cost specialized care such as chemotherapy, specialist palliative care, and renal dialysis [40]. The cost of the unbundled code was included in the total estimated cost for the episode. Episodes of care that could not be processed by the Cost Grouper due to missing data elements were assigned the average cost of episodes in the same diagnosis category. All episodes of care were assigned an appropriate cost based on the National Schedule of Reference Costs, and all episodes of care were summed to generate a total inpatient healthcare cost per patient. An average annual inpatient cost per patient was then derived by dividing by years of follow-up. Subjects with no hospital visits during the relevant time period were assigned an annual inpatient healthcare cost of zero.

### IV models

Three models were developed in this analysis to estimate the impact of BMI on inpatient healthcare costs.

1. A “naïve” regression that is a standard ordinary least squares regression of inpatient healthcare costs on observed BMI.
2. An instrumental variable model that uses the GRS instrument as a proxy for BMI to evaluate the association between inpatient healthcare costs and BMI.
3. An instrumental variable model with a two-part model of inpatient healthcare cost that uses the GRS instrument as a proxy for BMI, but accounts for the high proportion of subjects with zero inpatient healthcare costs.

The naïve, non-instrumental variable model was developed for comparison to the instrumental variable model. The regression of inpatient healthcare cost on BMI was adjusted for age, age squared, sex, ethnicity, socioeconomic status, smoking status, and the interaction of age and sex. Previous research has shown a U-shaped relationship between BMI and mortality, with underweight and obese patients at higher risk of death [41, 42]. A corresponding non-linear relationship between BMI and healthcare cost was also anticipated, so a BMI-squared term was also included in the regression. Age is also expected to have a non-linear relationship with BMI because healthcare costs tend to increase with age [43, 44]. Insignificant covariates were excluded from the final naïve model.

Several model types were tested in the development of the instrumental variable model. The first was a two-stage least squares (TSLS) model, which is the most common instrumental variable model used in MR studies [14]. The first stage of the TSLS model is a linear regression of BMI on the GRS and other covariates. The linear second stage regression predicts total inpatient healthcare costs from predicted BMI and the same covariates. An instrumental variable probit model was also tested to determine whether it would provide a better fit for the binary dependent variable. Best-fit models were chosen based on the F-statistic of the instrument and the magnitude of R^2^.

In addition to these standard instrumental variable models, two-part instrumental variable cost models were tested because a large proportion of patients incurred no healthcare costs. The first stage of the model was identical to the standard TSLS model. The second stage was constructed as a two-part model. The first part of the cost model was a logistic regression, which predicted whether subjects had non-zero costs. The second part of the cost model was an ordinary least squares or generalized linear model regression of predicted BMI on cost, conditional on the subject having non-zero costs. The generalized linear model used a log link and gamma distribution to account for the skewness of the cost data. The main outcome of all three models is the marginal effect of obesity on inpatient healthcare costs. In this analysis, marginal effects refer to the added incremental costs associated with obesity. All analyses were executed in STATA® version 14.

## Results

The mean BMI of the 482,127 subjects included in the analysis was 27.4 kg/m^2^. The average age of subjects was 56.5 years, 24% of subjects had BMI > 30 kg/m^2^, 94% were white, and 54% were female. Additional baseline characteristics are presented in Table 1.

**Table 1:** Baseline Characteristics

		Underweight [BMI < 18.5]	Normal [25 > BMI ≥ 18.5]	Overweight [30 > BMI ≥ 25]	Obese Class 1/2 [40 > BMI ≥ 30]	Obese Class 3 [BMI ≥ 40]	Total
		N (%)	N (%)	N (%)	N (%)	N (%)	N (%)
**Weighted GRS**	Mean (SD)	3.07 (0.53)	3.11 (0.52)	3.16 (0.52)	3.21 (0.52)	3.29 (0.52)	3.16 (0.52)
	Median (Q1, Q3)	3.06 (2.70, 3.42)	3.10 (2.75, 3.46)	3.15 (2.80, 3.51)	3.21 (2.86, 3.56)	3.29 (2.94, 3.65)	3.15 (2.80, 2.51)
**BMI t**_**0**_	Mean (SD)	17.6 (0.8)	22.9 (1.5)	27.3 (1.4)	33.1 (2.5)	43.6 (3.5)	27.4 (4.7)
	Median (Q1, Q3)	17.9 (17.3, 18.2)	23.1 (21.8, 24.1)	27.2 (26.1, 28.5)	32.5 (31.1, 34.6)	42.5 (41.1, 45.1)	26.7 (24.1, 29.9)
**Sex**	Male	514 (21%)	55,141 (35%)	109,199 (53%)	53,078 (49%)	2818 (32%)	220,750 (45.8%)
	Female	1980 (79%)	102,189 (65%)	96,208 (47%)	55,004 (51%)	5996 (68%)	261,377 (54.2%)
**Age**	Mean (SD)	55.4 (8.1)	55.7 (8.2)	57.0 (8.1)	60.0 (7.9)	55.6 (7.7)	56.5 (8.1)
	Median (Q1, Q3)	56 (49, 62)	57 (49, 63)	59 (51, 64)	58 (51, 63)	56 (50, 62)	58 (50, 63)
**Ethnicity**	White	2319 (93%)	149,143 (95%)	193,899 (94%)	101,231 (94%)	8157 (93%)	454,127 (94%)
	Black	10 (< 1%)	1427 (< 1%)	3063 (1%)	2613 (2%)	327 (4%)	7440 (2%)
	Mixed	20 (< 1%)	864 (< 1%)	941 (< 1%)	531 (< 1%)	52 (< 1%)	2408 (< 1%)
	Asian	84 (3%)	3895 (3%)	4643 (2%)	1937 (1%)	111 (1%)	10,670 (2%)
	Other	54 (2%)	1732 (1%)	2522 (1%)	1535 (1%)	141 (2%)	5984 (1%)
**Townsend Deprivation Index**	Mean (SD)	−0.7 (3.4)	−1.5 (3.0)	−1.4 (3.0)	− 0.91 (3.2)	− 0.02 (3.4)	−1.3 (3.1)
	Median (Q1, Q3)	−1.6 (−3.4, 1.7)	−2.3 (− 3.7, 0.2)	−2.3 (− 3.7, 0.3)	−1.76 (− 3.4, 1.2)	−.6 (− 2.8, 2.6)	−2.2 (− 3.7, 0.5)
	Missing	5	183	239	138	13	578
**Smoking Status**	Yes	570 (23%)	17,726 (11%)	20,949 (10%)	10,479 (10%)	810 (9%)	429,176 (10%)
	No	1911 (77%)	139,001 (88%)	183,417 (89%)	96,904 (90%)	7943 (90%)	50,534 (89%)
	Missing/Prefer Not to Answer	13 (< 1%)	603 (< 1%)	1041 (1%)	699 (< 1%)	61 (1%)	2417 (1%)
**TOTAL**		2494 (0.1%)	157,330 (32.6%)	205,407 (42.6%)	108,082 (22.4%)	8814 (1.8%)	482,127 (100%)
Estimated UK National Average^a^		< 1%	27%	39%	30%	4%	–

Health Survey for England 2017: Adult and child overweight and obesity. NHS Digital

^a^Adults aged 55–64 weighted by sex;

The instrument (GRS) was then checked for violation of any of the three instrumental variable assumptions (relevance, independence, exclusion-restriction). The F-statistic of our TSLS models were 3999 and 2453 for continuous and binary BMI respectively. Model F-statistics were well over the commonly used IV threshold of 10, which satisfies the relevance assumption [22, 32]. Linear regression of age, sex, and socioeconomic status found no significant relationship between the instrument and the confounding factors. The instrument was significantly associated with ethnicity (*p* < 0.001), which suggests a possible violation of the independence assumption by population stratification, which occurs when alleles occur with different frequencies in a population subgroup [22]. This finding is consistent with previous genetic studies that have shown varying effect sizes for obesity-related genes among different ethnicities [45, 46]. However, there was no significant association of ethnicity with inpatient healthcare cost (*p* = 0.896). This non-significance and the ethnically homogenous population (94% white) provide evidence against violation by population stratification. Assortative mating can also violate the independence assumption, though this cannot be directly tested. Genes are randomly assigned conditional on parental genes, but research suggests that individuals tend to select mates who are phenotypically similar to themselves [47–49]. This may violate the independence assumption if the mother and father’s genetic traits are associated with each other and with the outcome of inpatient healthcare costs [49].

Violation of the exclusion-restriction assumptions by linkage disequilibrium or pleiotropy cannot be directly tested. A search of NHGRI’s GWAS Catalog showed that some variants in the GRS may have multiple functions (pleiotropy) or be in linkage disequilibrium with variants associated with confounding factors or comorbidities. These variants were removed from the GRS in a sensitivity analysis and the direction of the estimated effect is the same as the primary analysis. The final naïve model is presented in Table 2.

**Table 2:** Naïve ordinary least squares regression results

Covariate	Continuous BMI					Binary BMI				
	Coefficient	SE	*P*-value	95% CI		Coefficient	SE	*P*-value	95% CI	
				Low	High				Low	High
BMI	21.611	0.652	< 0.001	20.333	22.889	205.527	7.183	< 0.001	191.449	219.604
Age	−49.341	5.492	< 0.001	−60.106	−38.577	−47.531	5.493	< 0.001	−58.297	−36.765
Age squared	0.619	0.050	< 0.001	0.522	0.716	0.608	0.050	< 0.001	0.511	0.705
Sex	− 644.472	43.352	< 0.001	− 729.441	− 559.502	− 606.837	43.339	< 0.001	−691.779	− 521.895
Sex and Age Interaction	11.591	0.758	< 0.001	10.104	13.077	11.155	0.758	< 0.001	9.669	12.641
Townsend index	8.979	1.034	< 0.001	6.952	11.005	9.544	1.034	< 0.001	7.518	11.571
Smoking Status	126.316	10.150	< 0.001	106.423	146.209	120.019	10.147	< 0.001	100.131	139.907
Black	104.144	25.287	< 0.001	54.583	153.706	116.879	25.284	< 0.001	67.324	166.435
Mixed	26.561	43.427	0.541	−58.555	111.676	25.539	43.440	0.557	−59.601	110.680
Asian	36.939	20.968	0.078	−4.157	78.036	32.292	20.972	0.124	−8.813	73.396
Other	30.554	28.049	0.276	−24.422	85.530	32.230	28.057	0.251	−22.761	87.222
Intercept	778.239	151.054	< 0.001	482.178	1074.300	1250.700	150.661	< 0.001	955.409	1545.990
R^2^	0.012					0.012				
Adjusted R^2^	0.012					0.012				

The association between healthcare cost and BMI was significant (*p* < 0.05). BMI-squared was also tested as the dependent variable in the naïve analysis because of the non-linear relationship between BMI and cost. However, using the squared term did not improve the model fit (R^2^), probably because so few (under 1%) of the study population were underweight (BMI < 18.5). The best-fit instrumental variable model was a TSLS model, presented in Table 3 for both continuous and binary BMI.

**Table 3:** Two-stage least squares regression results

	Covariate	Continuous BMI					Binary Obesity				
		Coefficient	SE	P-value	95% CI		Coefficient	SE	P-value	95% CI	
					Low	High				Low	High
STAGE 1	Weighted GRS	0.825	0.013	< 0.001	0.799	0.850	0.059	0.001	< 0.001	0.056	0.061
	Age	0.289	0.012	< 0.001	0.265	0.313	0.022	0.001	< 0.001	0.019	0.024
	Age squared	−0.002	0.000	< 0.001	−0.002	−0.002	0.000	0.000	< 0.001	0.000	0.000
	Sex	2.457	0.096	< 0.001	2.270	2.644	0.076	0.009	< 0.001	0.059	0.093
	Sex and Age Interaction	− 0.029	0.002	< 0.001	− 0.033	− 0.026	− 0.001	0.000	< 0.001	− 0.001	−0.001
	Townsend Index	0.145	0.002	< 0.001	0.140	0.149	0.012	0.000	< 0.001	0.012	0.013
	Current Smoker	−0.679	0.022	< 0.001	− 0.723	− 0.635	−0.041	0.002	< 0.001	−0.045	− 0.037
	Black	1.869	0.056	< 0.001	1.759	1.978	0.128	0.005	< 0.001	0.118	0.138
	Mixed	−0.008	0.096	0.934	−0.196	0.180	0.001	0.009	0.891	−0.016	0.018
	Asian	−0.630	0.046	< 0.001	−0.721	−0.540	− 0.050	0.004	< 0.001	− 0.058	− 0.042
	Other	0.352	0.062	< 0.001	0.231	0.473	0.025	0.006	< 0.001	0.013	0.036
	Intercept	15.539	0.335	< 0.001	14.883	16.195	−0.577	0.030	< 0.001	− 0.637	− 0.518
STAGE 2	BMI / Obesity	14.363	7.170	0.045	0.310	28.415	201.579	100.644	0.045	4.322	398.837
	Age	−47.242	5.869	< 0.001	−58.745	−35.739	−47.446	5.907	< 0.001	−59.023	−35.868
	Age squared	0.603	0.052	< 0.001	0.502	0.705	0.607	0.053	< 0.001	0.504	0.710
	Sex	− 626.552	46.813	< 0.001	−718.304	−534.800	−606.534	44.019	< 0.001	− 692.810	− 520.258
	Sex and Age Interaction	11.377	0.787	< 0.001	9.834	12.920	11.151	0.765	< 0.001	9.652	12.650
	Townsend Index	10.027	1.461	< 0.001	7.163	12.891	9.593	1.622	< 0.001	6.414	12.773
	Smoking Status	121.418	11.239	< 0.001	99.389	143.446	119.860	10.928	< 0.001	98.440	141.279
	Black	116.296	27.979	< 0.001	61.457	171.134	117.331	27.766	< 0.001	62.910	171.751
	Mixed	25.874	43.437	0.551	−59.262	111.010	25.520	43.442	0.557	−59.625	110.664
	Asian	30.997	21.772	0.155	−11.675	73.669	32.041	21.922	0.144	−10.925	75.007
	Other	32.182	28.098	0.252	−22.890	87.254	32.291	28.100	0.25	−22.784	87.366
	Intercept	909.619	198.924	< 0.001	519.735	1299.504	1249.149	155.737	< 0.001	943.911	1554.387
Model Fit	F-statistic	3999					2453				
	R^2^	0.031					0.017				
	Partial R^2^	0.008					0.005				

The relationship between predicted BMI and inpatient healthcare costs was significant (*p* < 0.05) in the TSLS model, but was not significant in the two-part cost models.

The naïve analysis of annual cost on continuous BMI predicts an additional healthcare cost of £21.61 [95% CI £20.33 – £22.89] associated with each additional BMI unit. The TSLS instrumental variable model predicts a slightly smaller cost difference of £14.36 [95% CI £0.31 – £28.42] per additional BMI unit. When the same models were executed with obesity expressed as a binary variable, the naïve analysis estimated £205.53 [95% CI £191.45 – £219.60] greater inpatient healthcare costs for obese subjects compared to non-obese subjects. The TSLS instrumental variable model found that inpatient healthcare costs were £201.58 [95% CI £4.32 – £398.84] greater for obese subjects compared to non-obese subjects. The two-part cost models did not find a significant difference in healthcare costs between obese and non-obese subjects, though the marginal effects were similar to the standard TSLS model. The predicted probability of being obese effectively ranges from 16 to 34% in the TSLS instrumental variable model. In the two-part cost model, the association between predicted BMI and inpatient healthcare cost is more significant (*p* = 0.003), and the odds of incurring hospital costs for obese patients is 1.02 [95% CI: 1.01–1.04] times that of non-obese patients. Marginal effects of each of these models are compared in Table 4.

**Table 4:** Model Comparison

Model	Continuous BMI					Binary Obesity				
	Marginal Effect	SE	*P*-Value	95% CI		Marginal Effect	SE	*P*-Value	95% CI	
				Low	High				Low	High
Naïve	21.611	0.652	< 0.001	20.333	22.889	205.527	7.183	< 0.001	191.449	219.604
TSLS	14.363	7.170	0.045	0.310	28.415	201.579	100.644	0.045	4.322	398.837
Two-Part Cost Model, OLS	15.312	12.112	0.206	−8.428	39.051	214.898	170.024	0.206	−118.345	548.140
Two-Part Cost Model, Gamma Family, Log Link	16.211	11.883	0.172	−7.079	39.500	227.519	166.773	0.172	−99.351	554.388

Note: *N* = 479,134 for first stage of all models; *N* = 287,776 in second part of two-part cost models

## Discussion

The results of both the TSLS and naïve models show significantly greater inpatient healthcare costs for obese patients in all versions of the model compared to non-obese patients. The impact of using instrumental variable methods compared to naïve models depends on whether BMI is expressed as a continuous or binary variable. When BMI was expressed as a binary categorical variable (above or below 30 kg/m^2^), the naïve and instrumental variable models estimated very similar marginal effects (£206 vs. £202), which suggests that the greater healthcare cost is almost entirely causal. When BMI was expressed as a continuous variable, the instrumental variable model estimated a smaller marginal effect than the naïve model (£14 vs £22), suggesting that only a portion of elevated costs are directly caused by greater BMI.

The IV model is more robust to residual confounding and reverse causality than the naïve model, but both models show very similar marginal effects. This similarity suggests that the naïve model is not strongly influenced by residual confounding and reverse causality. If the naïve model found a much larger cost difference between obese and non-obese individuals than the IV model, that would suggest that the cost difference estimated by the naïve model might be inflated by residual confounding. The naïve model has the advantage of a much tighter confidence interval than the IV model because the relationship between BMI and healthcare cost is estimated directly from the data instead of using GRS as a proxy.

In the TSLS model, predicted BMI is significantly (*p* < 0.05) associated with inpatient healthcare cost, but the *p*-value of 0.045 may be considered high given the large sample size. A large sample size is important to the strength of the instrument, and may reduce the likelihood of weak instrument bias, and offset the low explanatory power of the GRS [32, 50]. However, since 95% of the weighted GRS values are between 2.16 and 4.19, the predicted BMI effectively ranges between 26 and 29 kg/m^2^, which is close to the obesity threshold of BMI 30 kg/m^2^.

The MR approach used in this study makes the results less vulnerable to bias than standard regression analysis in scenarios where there is a high potential for unobserved confounding factors, and the large sample size reduces weak instrument bias [32, 50]. Results of both the instrumental variable and naïve models show a significantly greater inpatient cost for patients with BMI ≥30 kg/m^2^ than < 30 kg/m^2^ in all versions of the model, which is consistent with other studies of the impact of obesity on healthcare costs. A 2016 study of linked general practice and HES data found that annual healthcare costs were £456 [95% CI £344–£568] higher for subjects of BMI ≥ 40 kg/m^2^ [1]. An observational study by Tigbe et al. using data from the UK Counterweight Programme found an annual healthcare cost greater by £16 [95% CI: £11–£21] per unit BMI, when adjusted for age, sex, smoking status, alcohol intake, and physical activity [4]. Our analysis estimated a remarkably similar cost of £14.36 [95% CI £0.31 – £28.42] per additional unit of BMI, but in contrast our more recent analysis only focused on hospital expenditures, and did not assess total healthcare expenditures which have increased over the years.

A 2020 study by Dixon et al. (conducted at the same time as this study) undertook an MR analysis of linked UK Biobank and HES data, completely independently of the present analysis [21]. The 2020 study by Dixon and colleagues found marginal effects per additional unit of BMI ranging from £18.85 [95% CI: 9.05–28.65] to £21.22 [95% CI: 14.35–28.07] depending on the type of instrumental variable model used [21]. These results are not materially different from those presented in the current study, which found a marginal effect of £14.36 [95% CI £0.31 – £28.42] per additional unit of BMI. The minor differences in our results could be due to several factors. Dixon et al. included a greater number of alleles in the instrument, whereas the current study used an instrument with fewer alleles and had been generated and validated as a measure of obesity risk [31]. The F-statistic (a measure of the strength of the instrument) is higher in our study and suggests a stronger instrument, though the statistic is strongly influenced by sample size and it is likely a reflection of that factor [50]. The current study had the benefit of approximately two additional years of follow-up data and included approximately 100,000 more subjects. Both strategies produced instrumental variable analyses with F-statistics well above the threshold of 10 for a strong instrument. Both studies adopted the same costing methods, but used different versions of the NHS Cost Grouper software and national reference costs [21, 51]. Our study used the 2017/2018 Grouper, which has updated the valuation of procedure codes and included a larger net number of HRGs than the 2016/2017 Grouper [52].

The types of IV models chosen also differed between the studies. Our analysis included a standard TSLS model and a two-stage cost model similar to the one executed by Cawley and Meyerhoefer, while Dixon et al. implemented inverse variance weighted models and penalized weighted median models. Notably, our IV models found smaller effect sizes than the naïve models, except in the case of the two-part cost models using binary obesity. This is different than the results of Dixon [21] and Cawley and Meyerhoefer [19], where the opposite was true. Any combination of these differences covariates, sample size, and modelling strategy could be responsible for the differences in our results, but the fact that the results are similar in magnitude suggests that the results stand up to independent replication.

Only inpatient healthcare costs are considered in this analysis because outpatient, accident and emergency, and pharmacy data were not yet linkable to the UK Biobank data at the time the analyses were conducted. However, Tigbe et al. found that the positive association of healthcare cost with BMI extends beyond the inpatient setting. The study found that subjects with a BMI greater than 40 kg/m^2^ incurred significantly greater costs for prescription medication, primary care, and outpatient care than for subjects with a BMI less than 20 kg/m^2^ [4]. These additional costs are a subject for further research once additional ambulatory health record data is available for linkage with the UK Biobank.

This study also does not consider social care costs associated with obesity. In England, health and social care services cost about £17 billion per year, and about 70% of these annual costs are attributable to the care of individuals with long-term conditions [53, 54]. Obese individuals are at greater risk for diseases that require long-term care such as diabetes, cardiovascular disease, musculoskeletal disorders, and mental health disorders. These individuals often experience functional limitations and require long-term assistance with personal care, domestic tasks, transportation, housing and finances, which increases social care costs [53, 54]. A 2017 study by Copley et al. found that a 1 unit (kg/m^2^) increase in BMI was associated with a 5% increase in the odds of requiring social care. They also estimated that a BMI of 40 is associated with a nearly £500 increase in average annual social care cost compared to an individual with a normal BMI [52].

There are limitations to the generalizability of these results, including the ‘healthy volunteer’ bias of the UK Biobank data and the age limit of the patients recruited (40–69 years) [53]. Specifically, UK Biobank subjects were less likely to be obese, to report health conditions, or to smoke and drink [53, 55]. These individuals also had a lower mortality rate than the general population [55]. The non-representativeness of the UK Biobank sample introduces the potential for collider bias [56, 57]. In this case, the most severely obese individuals (who are also those at the highest genetic risk for obesity) are less likely to be represented in the data. If higher BMI and higher healthcare costs both reduce the likelihood of participation in the Biobank, an association is induced that violates the instrumental variable assumptions. Non-representative samples are common with large scale databases and this source of bias is typically small compared to other types of bias [56].

The costs reflect recent standard of care within the UK National Health Service, which may be different under other healthcare systems. Any MR analysis is limited by current knowledge of genes associations. The GRS used as an instrument in this analysis explained a significant but limited amount of variation in BMI. Future GWAS studies of obesity-related traits may reveal additional BMI-associated alleles that could explain more of the variation in BMI and create a stronger instrument. Unknown pleiotropy or linkage disequilibrium violations could also bias the analysis [13, 22]. Not all BMI-related alleles are necessarily related to body fat, given that they are derived from normal population among whom a half or more are likely to have BMI below 30, and thus influenced to a greater degree by variance in muscle mass, especially in younger people. The influence of parental behavior on their offspring may also violate the exclusion-restriction assumption. For example, a mother’s high-risk genotype may influence her behavior and preferences, which in turn may affect the child’s behavior either directly or through intrauterine effects of maternal adiposity [13, 58, 59]. If the mother’s genotype influences both the genotype of the child and the child’s inpatient healthcare cost, this represents a violation of the exclusion-restriction assumption [13, 19].

BMI is the most commonly used measure of obesity, but it is imperfect. BMI classification is based on a person’s height and weight, but does not distinguish between lean and fat mass. Therefore, BMI may be overestimated in people with higher muscle mass [35, 36]. Although there may be some cases where BMI is not a perfect measure, it is appropriate for use in this study because BMI was a common measure in the GWAS studies used to generate the GRS [26, 27]. There is however a potentially important source of error arising from the fact that the disease process of obesity (including its genetic elements) is present in large numbers of people before their BMI has risen to the conventional public health obesity threshold of 30 kg/m^2^. Thus, it is possible that some subjects in the sample who possess risk alleles may not yet have developed obesity or its comorbidities. Increase in size and number of fat cells triggers changes in metabolic and inflammatory processes, but comorbidities such as diabetes, atherosclerosis, osteoarthritis take time to develop [11, 60]. This may result in an underestimation of the costs attributable to obesity.

## Conclusions

The continuous and binary IV models provide evidence for a causal relationship between obesity and higher inpatient healthcare costs, independent of unobserved confounding factors such as lifestyle. Compared to the naïve model, the binary IV model found a slightly smaller marginal effect of obesity (₤201.58 vs. ₤205.53). The continuous IV model found a slightly smaller marginal effect of a single unit increase in BMI than the naïve model (₤14.36 vs. ₤21.61 annual cost). While we believe that this analysis is a very important step in understanding the role of endogeneity in determining causation in healthcare cost studies, further analyses with instrumental variables used in addition to weighted GRS would strengthen the results. At the time this analysis was conducted, general practice data were not yet linked to UK Biobank. Once the linked data become available, the MR analysis on all healthcare costs should be conducted to provide a more complete picture of healthcare costs attributable to obesity.

## Data Availability

The data that support the findings of this study are available from the UK Biobank but restrictions apply to the availability of these data, which were used under license for the current study, and so are not publicly available. Data are however available from the authors upon reasonable request and with permission of the UK Biobank.

## Abbreviations

MR: Mendelian Randomization
IV: Instrumental variable
HES: Hospital Episode Statistics
TSLS: Two-stage least squares
NHS: National Health Services
BMI: Body mass index
RCT: Randomized controlled trial
GWAS: Genome-wide association study
SNP: Small-nucleotide polymorphism
GRS: Genetic risk score
NHGRI: National Human Genome Research Institute
HRG: Healthcare Resource Group
SD: Standard deviation
